# Bullying at 8 years and violent offenses by 31 years: the Finnish nationwide 1981 birth cohort study

**DOI:** 10.1007/s00787-022-01964-1

**Published:** 2022-04-06

**Authors:** Elina Tiiri, Jaakko Uotila, Henrik Elonheimo, Lauri Sillanmäki, Anat Brunstein Klomek, Andre Sourander

**Affiliations:** 1 Department of Child Psychiatry, University of Turku, Turku University Hospital, Turku, Finland; 2 INVEST Research Flagship Center, University of Turku, Turku, Finland; 3 Department of Child Psychiatry, University of Turku, Turku, Finland; 4 Finnish Institute for Health and Welfare, Helsinki, Finland; 5 Baruch Ivcher School of Psychology, Interdisciplinary Center (IDC), Herzlyia, Israel

**Keywords:** Violence, Crime, Bullying, Victimization, Longitudinal studies

## Abstract

This study explored the associations between bullying perpetration and victimization at 8 years of age and violent offenses by the age of 31. Data were obtained for subjects enrolled in a population-based longitudinal birth cohort study. In 1989, 5813 8-year-old children (attrition 3.4%), and their parents and teachers, were surveyed about bullying. When 5405 subjects (attrition 10.2%) were 15–31 years of age, violent offenses were extracted from the Finnish National Police Register. We analyzed the data by sex and categorized bullying perpetration and victimization by frequency. Violent offenses were categorized by severity. Cox regression analyses estimated the hazard ratios (HRs) and 95% confidence intervals (95% CIs). When they were compared to males who had not been bullies at 8 years of age, frequent male bullies had an increased hazard for violent offenses (adjusted HR 3.01, 95% CI 2.11–4.33) and severe violent offenses (adjusted HR 2.86, 95% CI 1.07–7.59) as adults, even when the data were controlled for them being victims, parental education level, family structure and child psychopathology. Frequent female bullies also had an increased hazard for violent offenses, compared to those who had not bullied others (adjusted HR 5.27, 95% CI 1.51–18.40). Frequent male bullying was associated with higher odds for violent offenses compared to only bullying sometimes. Being a victim was not associated with violent offenses. Preventing childhood bullying could reduce violent offenses by both sexes.

**Supplementary Information:**

The online version contains supplementary material available at 10.1007/s00787-022-01964-1.

## Introduction

Bullying and violent offenses have an enormous global impact on the well-being of individuals and societies and preventing them is a key challenge for society. The American Centers for Disease Control and Prevention has recognized bullying as a form of violence and has defined it as unwanted repetitive aggressive behavior that takes place within an unequal power relationship and inflicts harm or distress on the victim [1]. Being bullied has been linked to students carrying out school shootings [2] and being a bully, a victim or both (a bully-victim) has been associated with adolescents carrying weapons [3].

Prospective studies have found that bullying perpetration in adolescence increases the risk of violence [4–6] but this has not been found for victimization [7]. Prospective population-based cohort studies on the association between bullying involvement in childhood and violent offenses in adulthood are scarce. These have found associations between being a bully and later violence [7–9]. Sourander et al. [10] studied this separately by sex and found significant associations for males, but not females, when the subjects were followed up from 23 to 26 years of age. This was based on the Finnish Nationwide 1981 Birth Cohort Study, one of the few prospective population-based cohort studies on the subject. In this cohort, victimization in childhood was not associated with later violent offenses at 16–20 [11, 12] and 23–26 years of age [10]. Being a bully [11, 12] or a bully-victim [12] was associated with violence among males at 16–20 years of age.

The present study was also based on the Finnish Nationwide 1981 Birth Cohort Study and it aimed to fill a gap in the literature by addressing some of the limitations of the previous studies. First, most longitudinal studies have gathered information about violent offenses using self-reports [7–9], which are vulnerable to underreporting or recall bias. The follow-up information in this study was based on police register data. Second, there has been a lack of studies on associations between childhood bullying and later violent offenses by females, although there have been differences in both bullying involvement [13] and committing violence [14, 15] between males and females. This association was assessed separately by sex in our previous study [10]. That study assessed violent offenses over a 4-years period, from 23 to 26 years of age, while the present study reports outcomes over a longer period, from the age of 15–31. Third, only two previous studies have provided information about childhood bullying and violent offenses until the age of 30 [7, 8]. They were based on the same birth cohort of 1265 children and the sample represented 78–79% of the surviving adult cohort. Our study adds to the literature by following up a large, representative sample, with a low attrition rate, until the age of 31. Fourth, previous longitudinal studies have not addressed how the frequency of bullying has been associated with the severity of violent offenses. Sourander et al. [10] reported an increase in the odds for violent offenses by males as the frequency of childhood bullying increased. Our study adds to this by addressing both the frequency of bullying and the severity of the violent offenses.

The first aim of this study was to examine the association between being a bully at 8 years of age and violent offenses by the age of 31, analyzed separately by sex. The second aim was to see if this association was stronger for those who were frequent childhood bullies than those who only bullied sometimes. Our third aim was to identify associations between bullying and severe violent offenses, including homicide. These were almost nonexistent among women and that is why these analyses were restricted to men. We hypothesized that there would be an association between being a bully and violent offenses in adulthood [7–10], but not between these offenses and being a victim of bullying [10]. Finally, we hypothesized that frequent childhood bullies would have higher odds for violent offenses compared to those who had only bullied sometimes.

## Methods

### Subjects and procedure

This study was part of the Finnish Nationwide 1981 Birth Cohort Study (Figure S1). The Joint Commission on Ethics of Turku University and Turku University Central Hospital approved the research plan. Participation was voluntary and based on informed consent from the children’s parents. The basic population was all 60,007 Finnish-speaking children born in Finland in 1981. At this time, 93.5% of people who lived in Finland, were Finnish-speaking [16]. A random sample of about 10% of the basic population was drawn, including rural, suburban and urban communities. The sample comprised 6017 children and 5813 (96.6%) took part. The baseline assessment was conducted in 1989, and information was obtained from the children, their parents and teachers [17]. Parents received information and questionnaires from the school via their child and returned their completed questionnaires to the teachers in a sealed envelope. The children filled in their questionnaires in the classroom and the teachers completed their questionnaires after parental consent. The teachers returned the study material to the research group.

### Information on bullying

Questions on bullying perpetration and victimization were included in the questionnaires [10–12, 18]. Children were asked to recall any bullying incidents during the past two weeks. Bullying was assessed by giving children three alternatives to choose from: “I bully other children almost every day”, “I bully sometimes” and “I do not usually bully”. Correspondingly, the alternatives to assess victimization were: “Other children bully me almost every day”, “Other children bully me sometimes” and “Other children do not usually bully me.” Parents and teachers were asked if the child had been a bully or a victim over the last 12 months and the responses were certainly applies, applies somewhat and does not apply*.* In the statistical analyses, the options indicated frequent involvement, some involvement or no involvement in bullying, respectively.

The analyses used pooled information from the children, parents and teachers. The perpetrators and victims were each split into three groups: no involvement, some involvement and frequent involvement, based on the highest rating of bullying by any informant. We also analyzed whether the associations between bullying and any violent offenses were different for bullies and bully-victims. Perpetrating bullying sometimes or frequently was recorded as being a bully or a bully-victim, and this depended on whether the subjects had also been victims of bullying. We formed a bullying variable with three categories for this additional analysis. The categories were those who had not bullied at all, but may have been victims of bullying, those who had just bullied and those who had been both bullies and victims of bullying.

### Covariates

The parents’ questionnaire included information on the child’s sex, parental education and family structure. The teachers’ questionnaire included information on the child’s psychopathology. These were used as covariates in the analyses. Parental education was based on whether or not one or both of them had completed upper secondary school education. The family structure options were living with two biological parents, one biological parent, one biological parent and a step-parent, foster parents, adoptive parents or some other family model. These were dichotomized for the statistical analyses into whether or not the child lived with two biological parents. Psychopathology was measured with the Rutter Teacher Questionnaire. This was reported to be more valid than the Rutter Parent Questionnaire when screening for psychiatric disturbances in the baseline sample of the study cohort [19] and was shown to have satisfactory reliability [20]. The Rutter Teacher Questionnaire includes an item on bullying perpetration and this item was removed for the analyses. The psychopathology scale was then dichotomized, with nine or more points indicating psychiatric problems. This was based on previous epidemiological studies [19, 20]. Because the bullying item was removed, we also conducted the analyses using eight points as the cut-off point to validate the results.

### Information on violent offenses

Information on violent offenses was obtained from the Finnish National Police Register, an electronic database that includes all cases where the police have suspected a named individual of an offense. The National Police Board provided permission. The registration threshold is low and if a person is suspected of multiple offenses, they all are registered. The data are archived after the window of time for prosecution has elapsed. Both the police register and the archive were examined to get comprehensive data that covered the follow-up period. The register data were linked with the study subjects by the personal identification code given to all Finnish citizens. This was approved by the Data Protection Ombudsman.

The age of criminal responsibility in Finland is 15 and that is why the observation period began at this age. The data were collected on 3 May 2012. The register includes the actual dates when the offenses were committed and this allowed us to monitor the follow-up period on a daily basis. Of the 5813 children who took part in the baseline study, seven had died and four had emigrated before they were 15 and the identification codes needed to link individuals to the police register were missing in 397 cases. We were able to check 5405 subjects (50.3% male) and this showed that 515 (9.5%) were registered for violent offenses, 36 had died and 95 had emigrated during the follow-up period. These 5405 subjects represented 89.8% of the original sample and 93.0% of those who participated in 1989. At the time of the data collection, 65.5% were aged 30 and 34.5% were 31.

The data on the crime register data included the crime category, which enabled us to identify violent offenses. Violent crime was defined as overt aggressive behavior toward another person and divided into minor and severe violent offenses (Table S1). The categorization was based on Finland’s Criminal Code. By dividing offenses into minor and severe violent offenses, we aimed to distinguish between the most severe forms of violent offenses from a legal point of view. For example, homicide was included in severe violent offenses. Attempted crimes were included in the respective groups.

### Statistical methods

The descriptive statistics of the study sample and violent offenses were calculated. The correlations between bullying perpetration and victimization, and the agreement between the informants, were calculated using dichotomic variables that indicated whether or not the child had been a bully or a victim. These were tested for the total sample, by including both males and females. We calculated the correlations using pooled information from the children, parents and teachers. Cohen’s kappa coefficient (κ) was used to calculate the level of agreement about bullying and victimization between the parents and children, the parents and teachers and the children and teachers. Attrition analysis was carried out for the sample that included both males and females. The characteristics of the study sample and the 1989 attrition group were compared. This included bullying perpetration and victimization, sex, whether or not the parents had completed upper secondary school education, the family structure and child psychopathology. The bullying perpetration and victimization variables were divided into three categories, as described above, and Pearson’s chi-square test was used to carry out the attrition analysis. The other variables were dichotomized and based on the coding described above. Fisher’s exact test was used to conduct these analyses.

Statistical models were created to predict any violent offenses and severe violent offenses. The explanatory variables were being a childhood bully or a victim. There were three categories that indicated involvement in bullying. These were never being a bully or a victim or being a bully or a victim sometimes or frequently. The separate sex × bully and sex × victim interactions for violent offenses were not significant, but we conducted separate sex-based analyses due to the differences in both bullying involvement [13] and violent criminality [14, 15]. Bullying was controlled for victimization and vice-versa. These variables were also divided into three categories that indicated involvement in bullying. These were never being a bully or a victim or being a bully or a victim sometimes or frequently. Parental education level, family structure and child psychopathology were also used as covariates. Hazard ratios (HRs) and 95% CIs were estimated using Cox regression analyses [21]. Survival time was defined as the amount of time that had elapsed from 15 years of age to the first event. The level of accuracy that was used to define time was 1 day, because the exact dates of the offenses were registered. The first event was the first violent offense of any severity when we analyzed any violent offenses. The first severe violent offense was used when we analyzed severe violent offenses. The subjects were censored at time of death or moving abroad or at the end of the study period if they had not been registered for offenses.

We carried out sensitivity analyses to separately estimate the associations between bullying perpetration or victimization at 8 years of age, as reported by the children, their parents and teachers, and any violent offenses by the age of 31. We used the dichotomic bullying and victimization variables to indicate whether or not the child had been a bully or a victim. The outcome variables fell into two categories and these were not committing any violent offenses, which was the reference category, or committing any violent offenses. Single predictor binary logistic regression models were carried out to estimate the odds ratios (ORs) and 95% confidence intervals (CIs).

We also carried out some additional analyses. First, the additional analyses assessed whether the OR for violent offenses in adulthood increased as the frequency of bullying perpetration at 8 years of age increased. The associations between more frequent male bullying and the severity of violent offenses were assessed using multinomial logistic regression analyses. The explanatory variable was bullying perpetration and this had three categories: bullying never, sometimes or frequently. Violent offending was the outcome variable and this was also divided into three categories. The reference category was males who had never committed any violent offenses and the other two categories were those who had only committed minor violent offenses or those who had committed severe violent offenses. If an individual had committed a number of offenses, we recorded the most serious offense for this analysis. ORs and 95% CIs were estimated.

Second, the additional analyses also assessed whether the HRs would be different for just bullies and bully-victims. The explanatory variable had three categories and these were no involvement in bullying as a perpetrator at 8 years of age, just being a bully at that age or being a bully-victim. The HRs and 95% CIs were estimated using Cox regression analyses. The first event was the first violent offense of any severity.

Two-sided *p* values of less than 0.05 were considered statistically significant, except for the interactions, which were based on 0.1. The statistical analyses were conducted using SAS 9.4 for Windows (SAS Institute Inc, Cary, NC, USA).

## Results

Table S2 presents the characteristics of the 5405 participants at baseline. There were slightly more boys (50.3%). Most children (83.7%) lived with two biological parents and 12.2% appeared to have psychiatric symptoms.

The correlation between bullying and victimization at 8 years of age was moderate, with a tetrachoric correlation coefficient of 0.52 (*p* < 0.001). Table S3 shows the cross-tabulation for the rates and frequencies of bullying and victimization. We found that the agreement about bullying and victimization between the parents and children, the parents and teachers and the children and teachers was rather low for both bullying (κ ranged from 0.19 to 0.26) and victimization (κ ranged from 0.12 to 0.23) (Table S4). It was notable that the children reported more victimization (34.5%) than their parents (23.5%) or teachers (11.6%). When it came to bullying, the reported rates were 21.4% by the children, 18.4% by their parents and 19.4% by their teachers. The results of the attrition analysis are presented in Table S5. The results were not statistically significant for bullying or victimization, sex and whether the parents had completed upper secondary school. However, the findings were statistically significant for family structure and child psychopathology and this indicated that a significantly larger proportion of children in the study sample lived with two biological parents and screened negative for psychopathology, compared to the attrition group.

Table 1 shows bullying frequency at 8 years of age and violent offenses by the age of 31. We found that 9.0% of the boys and 0.9% of the girls were frequent bullies and that 9.5% and 3.8% were frequent victims. Of the 405 men who had committed any violent offenses, 297 (73.3%) had been bullies, while the corresponding figure was 33 of the 81 women (40.7%). Of the 59 men who committed severe violent offenses, 48 (81.4%) had been bullies. The sex × bully interaction (*p* = 0.63) and the sex × victim interaction (*p* = 0.21) for violent offenses were not significant.

**Table 1:** Frequencies of males and females involved in bullying at eight years of age and violent offenses by the age of 31

	Males										Females									
	Total		Violent offenses								Total		Violent offenses							
			None		Minor		Severe		Homicide				None		Minor		Severe		Homicide	
	*n*	%	*n*	%	*n*	%	*n*	%	*n*	%	*n*	%	*n*	%	*n*	%	*n*	%	*n*	%
Bully																				
No	1124	43.8	1016	90.4	97	8.6	9	0.8	2	0.2	1911	75.8	1863	97.5	46	2.4	2	0.1	0	0.0
Sometimes	1213	47.2	996	82.1	182	15.0	28	2.3	7	0.6	589	23.4	560	95.1	28	4.8	0	0.0	1	0.2
Frequently	231	9.0	151	65.4	67	29.0	10	4.3	3	1.3	22	0.9	18	81.8	4	18.2	0	0.0	0	0.0
Victim																				
No	1097	42.8	946	86.2	135	12.3	14	1.3	2	0.2	1508	60.0	1464	97.1	42	2.8	2	0.1	0	0.0
Sometimes	1224	47.7	1025	83.7	166	13.6	26	2.1	7	0.6	909	36.2	873	96.0	35	3.9	0	0.0	1	0.1
Frequently	243	9.5	186	76.5	46	18.9	8	3.3	3	1.2	95	3.8	94	99.0	1	1.1	0	0.0	0	0.0

Table S6 shows the distribution of violent offenses by the frequency of bullying in childhood. The 9.0% of the men who were frequent bullies in childhood committed 25.1% of all the violent offenses related to the cohort in adulthood. When it came to the women, the 0.9% who were frequent bullies in childhood committed 5.3% of all the violent offenses related to the cohort in adulthood. As a whole, the 5.0% of the children who were frequent bullies committed 23.1% of the violent offenses.

Table 2 shows the HRs for any violent offenses. When they were compared with the men who had not been bullies, there were statistically significant findings for men who were bullies sometimes (HR 1.92, 95% CI 1.50–2.45) or frequently (HR 3.76, 95% CI 2.71–5.20) in childhood, when the data were controlled for victimization, parental education levels and family structure. When child psychopathology was also controlled for, the hazards were still increased for those who bullied sometimes (HR 1.84, 95% CI 1.44–2.35) or frequently (HR 3.01, 95% CI 2.10–4.33). The hazard was also increased in women for both being a bully sometimes (HR 1.87, 95% CI 1.15–3.04) and frequently (HR 7.28, 95% CI 2.20–24.13), when controlled for victimization, parental education levels and family structure. The reference group were the women who had not been bullies. When the data for women were further controlled for child psychopathology, the hazards were still higher for both being a bully sometimes (HR 1.73, 95% CI 1.05–2.86) and frequently (HR 5.27, 95% CI 1.51–18.40). Men who were frequent victims showed increased hazards for violent offenses in the unadjusted model (HR 1.82, 95% CI 1.34–2.47), compared to men who had not been victimized by bullies. When this was controlled for those who were also perpetrators, there were no statistically significant findings. There were no statistically significant findings for victimization among the women.

**Table 2:** Bullying perpetration and victimization at eight years of age and committing any violent offenses by 31 years of age. Summary of Cox regression models

	Any violent offenses			
	Unadjusted HR (95% CI)	Adjusted HR (95% CI)^a^	Adjusted HR (95% CI)^b^	Adjusted HR (95% CI)^c^
Bully				
Males				
No	1	1	1	1
Sometimes	1.95 (1.55 − 2.45)***	1.96 (1.55 − 2.49)***	1.92 (1.50–2.45)***	1.84 (1.44 − 2.35)***
Frequently	4.33 (3.24 − 5.78)***	4.19 (3.06 − 5.74)***	3.76 (2.71–5.20)***	3.01 (2.10 − 4.33)***
Females				
No	1	1	1	1
Sometimes	1.99 (1.25 − 3.15)**	1.98 (1.21 − 3.22)**	1.87 (1.15–3.04)*	1.73 (1.05 − 2.86)*
Frequently	7.88 (2.84 − 21.86)***	8.09 (2.44 − 26.77)***	7.28 (2.20–24.13)**	5.27 (1.51 − 18.40)**
Victim				
Males				
No	1	1	1	1
Sometimes	1.20 (0.97 − 1.48)	0.93 (0.75 − 1.17)	0.86 (0.69–1.08)	0.83 (0.66 − 1.04)
Frequently	1.82 (1.34 − 2.47)***	1.11 (0.79 − 1.54)	0.98 (0.69–1.37)	0.89 (0.63 − 1.27)
Females				
No	1	1	1	1
Sometimes	1.37 (0.88 − 2.12)	1.11 (0.69 − 1.77)	1.09 (0.68–1.74)	1.04 (0.64 − 1.67)
Frequently	0.36 (0.05 − 2.60)	0.23 (0.03 − 1.72)	0.24 (0.03–1.74)	0.20 (0.03 − 1.50)

Note: *p < 0.05; **p < 0.01; ***p < 0.001. *HR* hazard ratio

^a^Adjusted for perpetration (when analyzing victimization) or victimization (when analyzing perpetration)

^b^Adjusted for perpetration or victimization and family variables (parental education level and family structure)

^c^Adjusted for perpetration or victimization, family variables and child psychopathology (the bullying question was removed)

The association between childhood bullying and severe violent offenses was studied among men (Table 3). The unadjusted model showed that the HR for frequent bullies (HR 6.55, 95% CI 2.87–14.94) was considerably higher than among those who had bullied sometimes (HR 3.28, 95% CI 1.62–6.62). In the adjusted models, the HR maintained this increase for both frequent bullies (HR 4.67, 95% CI 1.90–11.49) and those who bullied sometimes (HR 2.82, 95% CI 1.37–5.82), when controlled for victimization, parental education level and family structure. When the data were further controlled for child psychopathology, the HRs were still higher for frequent bullies (HR 2.86, 95% CI 1.07–7.59) and those who bullied sometimes (HR 2.50, 95% CI 1.20–5.21). The reference group were the men who had not been bullies. When it came to victimization, the unadjusted model showed increased HRs for severe violent offenses for both those who were victims sometimes (HR 1.87, 95% CI 1.03–3.39) and frequently (HR 2.88, 95% CI 1.31–6.34), compared with the men who had not been bullied. When these were controlled for them also being perpetrators, the findings became nonsignificant. In the analyses presented in Tables 2 and 3, scoring nine or more points for the Rutter Teacher Questionnaire indicated psychiatric problems. Because the question on bullying perpetration had been removed from the Rutter Teacher Questionnaire, we used a cut-off point of eight to conduct the analyses to validate the results. This did not alter the significance of the results in any of the Cox regression analyses.

**Table 3:** Bullying by boys at eight years of age and committing severe violent offenses, including attempted or actual aggravated assault, manslaughter and murder, by 31 years of age. Summary of Cox regression models

	Severe violent offenses			
	Unadjusted HR (95% CI)	Adjusted HR (95% CI)^a^	Adjusted HR (95% CI)^b^	Adjusted HR (95% CI)^c^
Bully				
No	1	1	1	1
Sometimes	3.28 (1.62 − 6.62)***	3.01 (1.46 − 6.19)**	2.82 (1.37 – 5.82)**	2.50 (1.20 − 5.21)*
Frequently	6.55 (2.87 − 14.94)***	5.82 (2.42 − 13.99)***	4.67 (1.90 – 11.49)***	2.86 (1.07 − 7.59)*
Victim				
No	1	1	1	1
Sometimes	1.87 (1.03 − 3.39)*	1.34 (0.72 − 2.47)	1.23 (0.66 – 2.28)	1.12 (0.60 − 2.09)
Frequently	2.88 (1.31 − 6.34)**	1.44 (0.61 − 3.45)	1.37 (0.57 – 3.28)	1.11 (0.46 − 2.70)

Note: *p < 0.05; **p < 0.01; ***p < 0.001. *HR* hazard ratio

^a^Adjusted for perpetration (when analyzing victimization) or victimization (when analyzing perpetration)

^b^Adjusted for perpetration or victimization and family variables (parental education level and family structure)

^c^Adjusted for perpetration or victimization, family variables and child psychopathology (the bullying question was removed)

**Fig. 1:**
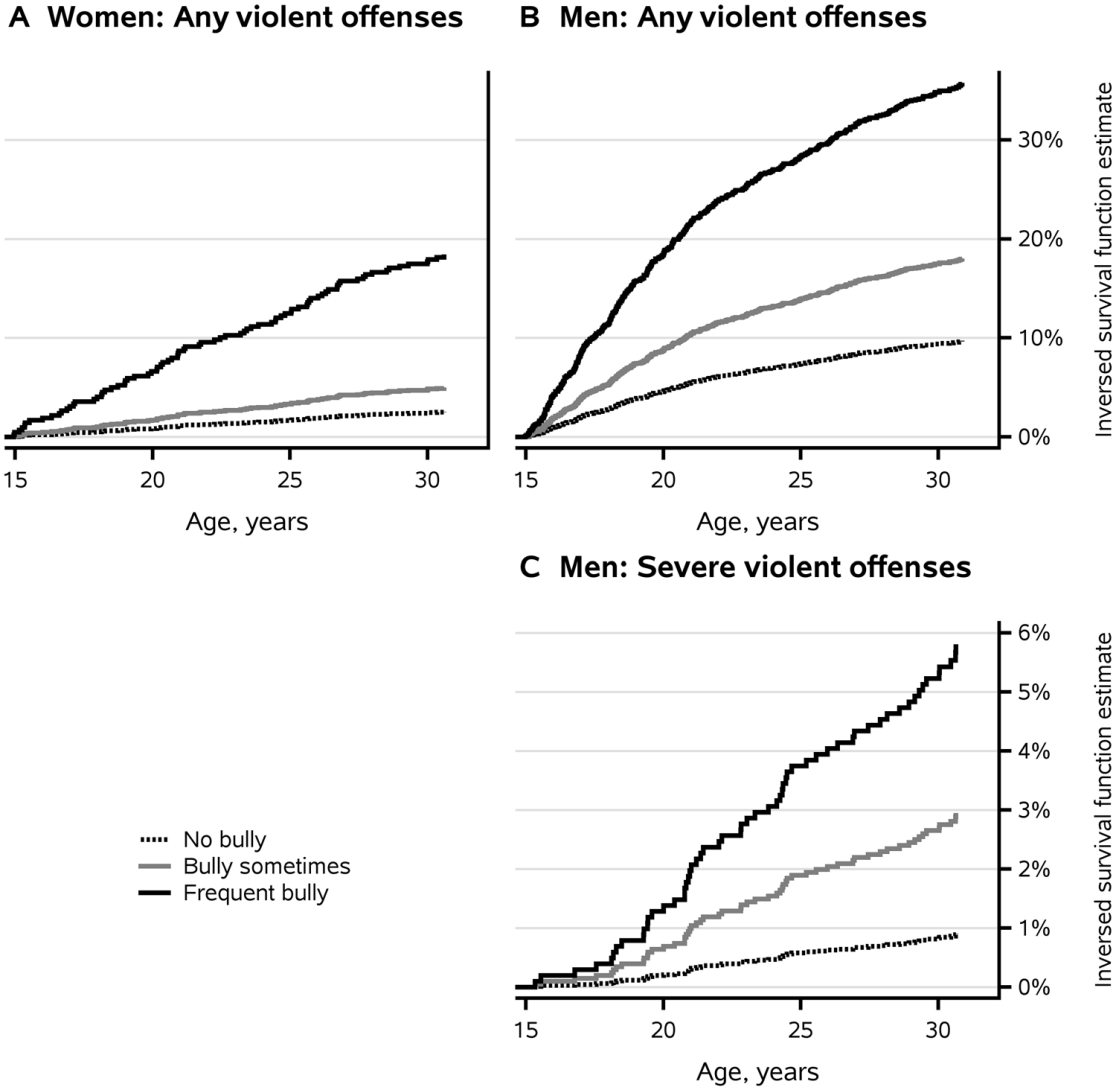
Inverse survival function estimates of the first violent offense by age: **A** any violent offenses by women who bullied frequently, sometimes or did not bully, **B** any violent offenses by men who bullied frequently, sometimes or did not bully and **C** severe violent offenses, including homicide, by men who bullied frequently, sometimes or did not bully. Cox regression model, unadjusted. Table S7 shows the numbers of individuals at risk for committing violent offenses at the ages of 15, 20, 25 and 30 years, presented separately for **A**, **B** and **C**

**Fig. 2:**
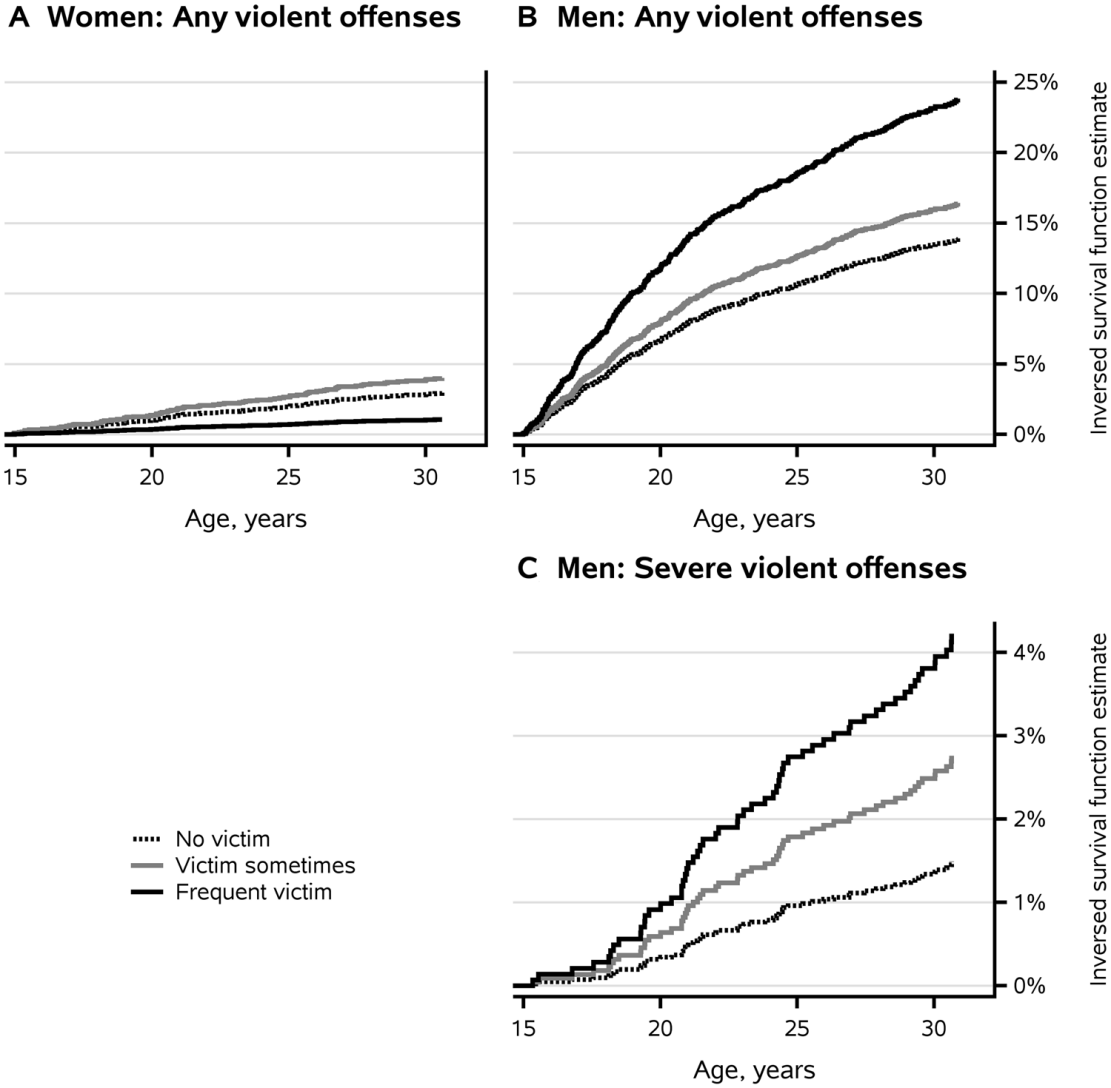
Inverse survival function estimates of the first violent offense by age: **A** any violent offenses by women who were bullied frequently, sometimes or were not bullied, **B** any violent offenses by men who were bullied frequently, sometimes or were not bullied and **C** severe violent offenses, including homicide, by men who were bullied frequently, sometimes or were not bullied. Cox regression model, unadjusted. Table S7 shows the numbers of individuals at risk for committing violent offenses at the ages of 15, 20, 25 and 30 years, presented separately for **A**, **B** and **C**

Figure 1 shows the inverse survival function estimates for any violent offenses by men and women and for severe violent offenses by men, based on the unadjusted Cox regression models. The predictor was bullying others. Based on the figure, the hazard for any violent offenses over time among men showed the greatest increase until the age of just over 20 years. The hazard was highest for frequent bullies. Similarly, the inverse survival function estimates in Fig. 2 were based on the unadjusted Cox regression models. Being a victim of bullying was the predictor for any violent offenses by men and women and for severe violent offenses by men. Table S7 shows the numbers of individuals who risked committing violent offenses and this data corresponds to Figs. 1 and 2.

The findings of the sensitivity analyses (Table S8), which were carried out separately for each informant, namely the children, their parents and teachers, were comparable to the findings of the pooled analyses for all of the informants. In other words, being a bully at 8 years of age predicted committing any violent offenses by 31 years of age, but victimization by bullying did not. The finding of the self-reports by female bullies was marginal (*p* = 0.050). Being a frequent bully during childhood was associated with larger odds for violent offenses by men compared to being a bully sometimes (Table S9). More specifically, when frequent bullies were compared to those who bullied sometimes, there were higher odds for both minor (OR 2.43, 95% CI 1.75–3.37) and severe (OR 2.45, 95% CI 1.27–4.74) violent offenses in the unadjusted analyses. We also assessed whether bullies and bully-victims had different HRs for any violent offenses (Table S10). While both bullies and bully-victims had increased hazard for violent offenses, compared to those who did not bully, there were no statistically significant findings among men or women when bully-victims were compared to bullies.

## Discussion

Our study had three key findings. First, being a bully at the age of eight was associated with any violent offenses by men and women in adulthood. The findings remained significant after adjusting for victimization by bullying, parental education level, family structure and child psychopathology. Second, severe violent offenses were strongly associated with frequent bullying among men, although the associations were considerably weaker after we controlled the data for child psychopathology. Third, we found that males who frequently bullied others when they were 8 years old were more strongly associated with violent offenses as adults, compared to those who bullied only sometimes.

The strong association between childhood bullying perpetration and severe violent offenses among men was a striking finding. So was the finding that being a bully at the age of eight was associated with any violent offenses among both sexes. Previous prospective studies have reported associations between bullying perpetration and violence [4–12]. However, our study broadened this understanding by showing associations in females and by analyzing the severity of violent offenses. Research has also shown that the prevalence of crime tends to peak in late adolescence and then decreases in adulthood [22, 23]. We found that the hazard for the first violent offense by men showed the greatest increase until just over 20 years of age. We found that controlling for parental education level and family structure reduced the hazard associated with bullying. This was in line with the findings of previous studies, which indicated associations between bullying and socioeconomic adversities [8, 24]. A previous report based on the Finnish Nationwide 1981 Birth Cohort Study indicated the impact that mental health symptoms may have on future criminal offenses. It stated that males who had been frequent bullies, and had displayed mental health symptoms, were more than three times as likely to commit criminal offenses in late adolescence than those who were not bullies and did not have any symptoms. Interestingly, the odds did not differ between frequent bullies and those who had not been bullies, when these groups did not have any mental health symptoms [12]. In our study, controlling for psychopathology also reduced the hazard associated with bullying, especially among frequent bullies. These findings add to the literature on the impact that psychopathology has on the association between bullying and profound consequences [25]. In this case, controlling for psychiatric symptoms weakened the risk that children who bullied others would commit violent offenses later in life. Bullies have been found to demonstrate proactive aggression [13] and show high levels of callous-unemotional traits [26], characterized by low empathy and guilt [27]. Bullying and violent offenses may have the same underlying antisocial or violent dispositions, but display different behavioral manifestations during different developmental periods. Farrington and Ttofi [5] suggested that interventions that decrease bullying might decrease later violent offending. Our findings support previous theories that bullying, combined with psychopathology, should be targeted by early interventions that address both bullying and mental health problems. Multicomponent, evidence-based interventions should be implemented to reduce the factors that increase the risk of later violence [28, 29]. These can include programs that focus on parental training, cognitive behavioral skills and positive mental health, as well as those that focus on reducing deviant behavior in different environments.

Our findings showed that being a frequent childhood bully was strongly associated with violent offenses as adults. First, males who had frequently bullied other children had the strongest associations with violent adult offenses. Second, this was also found between male bullies and men who committed severe violent offenses. Third, when frequent male bullies were compared with those who only bullied sometimes, their odds for both minor and severe violent offenses were higher in the unadjusted analyses. This persisted for minor violent offenses in the adjusted analyses. These findings on violent offenses may be explained by some frequent bullies having traits and risk factors that predispose them to continuing childhood aggression into adulthood, such as callous-unemotional traits [26]. Previous studies have reported that those who had been chronically bullied were more likely to have psychotic symptoms [30] and poorer social relationships and wealth [31] later in life than those who were only bullied at one point in their life. However, there have not been any studies that have focused on the effects that being a bully has on violence. Nevertheless, frequent bullying in childhood has been reported to have stronger associations with reoffending in young adulthood than infrequent bullying [10].

We found that both bullies and bully-victims had an increased hazard for violent offenses, when they were compared to people who had not bullied others. However, when we assessed the HR for violent offenses among bully-victims, we found that it did not differ from that of pure bullies. This finding was somewhat unexpected. Bully-victims form a distinct group as early as childhood and they are characterized by dysregulation, mental health problems and both proactive and reactive aggression [13]. Longitudinal studies have reported that bully-victims, in particular, are at risk of negative long-term outcomes [32], even compared to pure bullies. These outcomes include having an antisocial personality disorder [18], which is characterized by aggressive behavior. On the other hand, there have been hardly any longitudinal studies on whether being a bully-victim has led to violence as a long-term outcome. Our study was based on the same cohort as the only previous report to study the associations between being a childhood bully-victim and committing violent offenses later in life. That study found that males who had been bully-victims in childhood had higher odds for violent offenses at 16–20 years of age, compared to males who had not been involved in bullying [12]. Our present study adds to the literature, by showing that both male and female childhood bully-victims had an increased hazard for violent offenses, when they were compared to those who had not been bullies. It also shows that they had the same hazards as people who bullied others, but were not victims.

Another important finding was the association between childhood bullying and adult women committing violent offenses. Only two longitudinal studies have reported the association between bullying and later criminal [10] or antisocial [33] outcomes separately by sex. These reported stronger associations for adverse outcomes among men than women. Our previous study did not find any association between bullying and later violent offenses by females at the age of 23–26, but it focused on a smaller number of cases, due to the shorter follow-up interval than the present study [10]. In general, studies on childhood predictors of violence have focused on males [11, 12, 29]. This may be because direct aggression, particularly physical aggression, is less common among females than males from early childhood into adulthood [34]. A developmental trajectory study reported that girls who were chronically aggressive in childhood did not have a similar risk for delinquency in adolescence as boys. This may indicate some protective factors [35] or fewer risk factors for girls [36]. On the other hand, the much rarer female offending has been argued to indicate more deviance than male offending [37]. Previous studies have reported a higher rate of many disorders [38] and mortality [37, 38] among female offenders than male offenders. Our baseline study was conducted in 1989 and it is possible that the main focus was on physical bullying at the time. Thus, it is possible that not all children using relational tactics to bully were recognized as bullies and these were more likely to be girls [13]. Despite this, our study showed that there were similar associations between childhood bullying and later violent offenses between men and women.

Being bullied was not associated with later violent offenses. In the unadjusted analysis, frequent male victims had an increased HR, but this did not persist when we controlled for whether they were also perpetrators. Similarly, previous studies on being bullied in childhood [10, 12] or adolescence [7] did not find increased odds for later violence. One study suggested that some school shootings were violent retaliation by people who had been bullied and that they could have represented provocative aggression [2]. However, our study findings point to the association between being a bully as a child and criminal activity as an adult. Victimization was more likely to have been associated with interpersonal difficulties, such as low confidence in social interaction and internalizing problems in both childhood [13] and adulthood [32].

The strengths of the study included the large and representative, population-based sample, three informants at baseline, the use of official police data and the prospective study design with a long follow-up interval. The police register data included the dates when the suspected crimes were committed. This allowed us to use Cox regression analysis, which was a novel approach to studying the association between childhood bullying involvement and later violence. However, there were also some limitations. First, the initial 1989 questionnaire did not provide a definition of bullying. There was one question each on bullying and victimization and the types of traditional bullying were not explored. However, the questions provided in the initial questionnaire did not just predict long-term criminal outcomes [10–12]. They also pointed to long-term mental health disorders [18]. Furthermore, a study reported that 8-year-olds can see the difference between aggressive and nonaggressive behavior, but do not distinguish between forms of aggression as clearly [39]. Second, although the attrition rate at the initial data collection was low in 1989 (3.4%), 10.2% of the register data were missing. This was due to a non-systematic error in the initial data collection, as some of the subject’s personal identification codes were not documented and the link with the police register could not be established. However, it is not likely that the attrition rate would have induced a major bias, because the overall attrition rate was low and the findings of the attrition analysis were not significant for bullying perpetration and victimization. Third, any source of information on violent offenses contained possible bias due to the nature of the subject. However, we used the Finnish National Police Register and false positives were unlikely to be a source of bias, due to strict legislative control over the police and the fact that Finland is one of the least corrupt countries in the world. Although not all violent offenses are detected, and registered, using this source of information increases the reliability of research compared to other methods. For example, court data on convictions is likely to reflect fewer violent offenses than the low-threshold Finnish National Police Register and methods that use self-reports could be subjected to recall bias or a reluctance to reveal violent acts. In addition, being able to obtain data on violent offenses to within an accuracy of one day allowed us to monitor the study population carefully over the follow-up period.

## Conclusion

Bullying and violent offenses may indicate a similar underlying tendency for violence, with various behavioral manifestations in different developmental periods. However, it is important to take a broader view of preventing bullying and consider it a possible way of preventing future violence [5, 29]. We found that the associations between bullying and violent offenses were weaker when child psychopathology was controlled for and this was supported by previous research [5, 7, 8, 10]. That is why it is important to actively integrate mental health promotion and treatment with bullying prevention. Because the adverse effects of bullying extend beyond violence [32, 40], any bullying prevention program should cover all those involved in bullying as bullies, victims or bully-victims.

## Supplementary Information

Below is the link to the electronic supplementary material.

Supplementary file1 (PDF 247 KB)

## Data Availability

Not applicable.

## Declarations

### Software application

SAS 9.4 for Windows (SAS Institute Inc. Cary, NC, USA, 2012).
