# Supplementary material for: Bullying at 8 years and violent offenses by 31 years: the Finnish nationwide 1981 birth cohort study
Source: Eur Child Adolesc Psychiatry. 2022 Apr 6;32(9):1667–78. doi: 10.1007/s00787-022-01964-1 (PMC10460349; doi:10.1007/s00787-022-01964-1)
Supplement: Supplementary file 1 — Supplementary file1 (PDF 247 KB) [file 787_2022_1964_MOESM1_ESM.pdf]

Bullying at eight years and violent offenses by 31 years: the Finnish Nationwide 1981 Birth Cohort Study

European Child & Adolescent Psychiatry

Tiiri Elina, Uotila Jaakko, Elonheimo Henrik, Sillanmäki Lauri, Brunstein Klomek Anat, Sourander Andre

Corresponding author: Andre Sourander, andsou@utu.fi

Department of Child Psychiatry, University of Turku and Turku University Hospital, Turku, Finland and INVEST Research Flagship Center, University of Turku, Turku, Finland.

Fig. S1 Flow chart showing the participants in the study

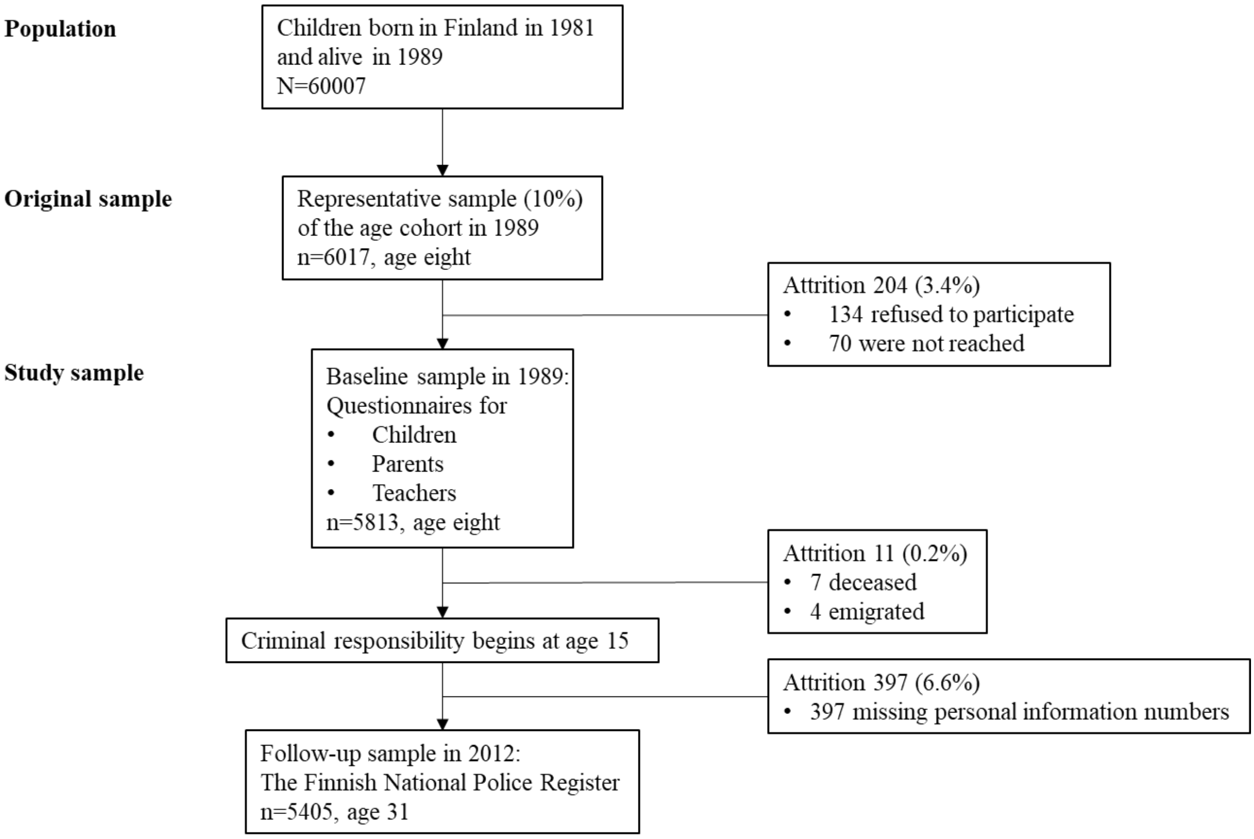

Table S1 Violent offenses considered as minor and severe in this study

Minor:	Abandonment	
	Assault, basic	
	Assault, minor	
	Assault, attempted	
	Coercion	
	Deprivation of liberty	
	Resistance to a person maintaining public order	
	Resistance to a public official	
	Resistance to a public official, violent	
	Robbery, basic	
	Robbery, attempted	
	Robbery, aggravated	
	Robbery, attempted aggravated	
	Taking part in a fight	
	Threatening a person to be heard in the administration of justice	
	Violent behavior in a public vehicle	
	Violent behavior toward a guard	
	Violent threatening	
Severe:	Aggravated assault	
	Aggravated assault, attempted	
	Homicide:	Manslaughter
		Manslaughter, attempted
		Murder (no cases in this study)
		Murder, attempted

Note: The aim of the categorization was to distinguish between the most severe forms of violent offenses from a legal point of view. This does not imply that those defined as minor in this study would be considered minor from a moral perspective or by the victims. Robbery was included in minor violent offenses, because it may have involved only threats of violence. Furthermore, aggravated robbery was categorized as minor, because it may have only involved showing, but not using, a weapon.

Table S2 Background characteristics of the baseline sample of children at eight years of age

	n	%
Sex		
Male	2718	50.3
Female	2687	49.7
Mother completed upper secondary school		
Yes	1501	29.7
No	3558	70.3
Father completed upper secondary school		
Yes	1008	21.7
No	3628	78.3
Family background		
Two biological parents	4339	83.7
One biological parent	517	10.0
One biological parent and a step-parent	272	5.2
Foster parents	21	0.4
Adoptive parents	14	0.3
Other	20	0.4
Psychopathology of the child		
Screen negative	4642	87.9
Screen positive	642	12.2

Note: Nine or more points on the Rutter Teacher Questionnaire indicated psychiatric problems

Table S3 Cross-tabulation between bullying perpetration and victimization at eight years of age

	Victim			
	No		Yes	
	n	%	n	%
Bully				
No	1972	39.1	1044	20.7
Yes	623	12.3	1410	27.9

Table S4 Agreement about bullying and victimization between the parents and children, the parents and teachers and the children and teachers

	Bully	Victim
	κ	κ
Parent / child	0.19	0.19
Parent / teacher	0.25	0.23
Child / teacher	0.26	0.12

Note: κ kappa coefficient

Table S5 Attrition analysis that compared the background characteristics of the study sample and the attrition group in 1989

	Study sample		Attrition		
	n	%	n	%	*p* value
Bully					0.83[a]
No	2952	59.6	352	60.8	
Sometimes	1758	35.5	200	34.5	
Frequently	247	5.0	27	4.7	
Victim					0.18[a]
No	2538	51.4	287	49.5	
Sometimes	2078	42.1	243	41.9	
Frequently	326	6.6	50	8.6	
Sex					0.20[b]
Male	2653	51.0	294	48.2	
Female	2551	49.0	316	51.8	
Mother completed upper secondary school					0.41[b]
Yes	3474	70.5	392	66.8	
No	1453	29.5	178	31.2	
Father completed upper secondary school					0.46[b]
Yes	3545	78.4	406	79.9	
No	976	21.6	102	20.1	
Family background					0.0050[b]
Two biological parents	4225	83.9	467	79.2	
Other	814	16.2	123	20.9	
Psychopathology of the child					0.0063[b]
Screen negative	4521	87.9	504	83.9	
Screen positive	625	12.2	97	16.1	

[a] Pearson's chi-squared test

[b] Fisher’s exact test

Table S6 The distribution of violent offenses by the age of 31 by the frequency of bullying perpetration in childhood

	Violent offenses													
	Males							Females						
	n	%	Mean	SD	Median	Min	Max	n	%	Mean	SD	Median	Min	Max
Bully														
No	235	17.7	0.21	0.95	0	0	14	82	54.7	0.04	0.34	0	0	7
Sometimes	762	57.3	0.63	2.33	0	0	31	60	40.0	0.10	0.62	0	0	11
Frequently	334	25.1	1.45	3.82	0	0	31	8	5.3	0 36	0.95	0	0	4

Note: *SD* standard deviation

Table S7 The numbers of individuals at risk for committing violent offenses at ages 15, 20, 25 and 30. This data corresponds to Figures 1 and 2.

				Age			
				15	20	25	30
				n	n	n	n
Bully	Females	Any violent offenses	No bully	1911	1887	1857	1815
			Bully sometimes	588	578	565	543
			Frequent bully	22	21	20	19
	Males	Any violent offenses	No bully	1124	1079	1036	1000
			Bully sometimes	1214	1106	1040	985
			Frequent bully	231	183	163	151
		Severe violent offenses	No bully	1123	1122	1113	1101
			Bully sometimes	1214	1208	1182	1158
			Frequent bully	231	227	218	215
Victim	Females	Any violent offenses	No victim	1501	1488	1462	1426
			Victim sometimes	907	892	876	848
			Frequent victim	95	95	94	94
	Males	Any violent offenses	No victim	1097	1023	979	933
			Victim sometimes	1225	1126	1060	1015
			Frequent victim	243	216	196	182
		Severe violent offenses	No victim	1097	1092	1083	1065
			Victim sometimes	1225	1218	1192	1169
			Frequent victim	242	241	233	229

Table S8 Sensitivity analysis carried out separately for each informant, namely the children, their parents and teachers, on the association between bullying perpetration and victimization at eight years of age and committing any violent offenses by 31 years of age. The results of single predictor binary logistic regression models.

	Any violent offenses	
	Males	Females
	Unadjusted OR (95% CI)	Unadjusted OR (95% CI)
Bully		
Child	1.92 (1.54–2.40)***	1.75 (1.00–3.08)[a]
Parent	1.50 (1.17–1.93)**	3.03 (1.65–5.58)***
Teacher	2.71 (2.15–3.41)***	2.72 (1.39–5.33)**
Victim		
Child	1.05 (0.84–1.30)	1.04 (0.65–1.69)
Parent	1.10 (0.85–1.41)	0.80 (0.43–1.51)
Teacher	1.01 (0.77–1.33)	0.96 (0.40–2.29)

Note: *p < 0.05; **p < 0.01; ***p < 0.001. *OR* odds ratio. The reference group for bullying were those who were not bullies, and for victimization, those who were not victims.

[a] p=0.050

Table S9 The association between the increasing intensity of bullying perpetration at eight years of age and violent offenses by males. Severe violent offenses include homicide.

Bullying perpetration	Minor violent offenses		Severe violent offenses	
	Unadjusted OR (95% CI)	Adjusted OR (95% CI)[a]	Unadjusted OR (95% CI)	Adjusted OR (95% CI)[a]
Sometimes vs. never	1.91 (1.47–2.49)***	1.86 (1.40–2.47)***	3.25 (1.64–6.43)***	2.42 (1.18–4.96)*
Frequently vs. never	4.65 (3.26–6.63)***	3.43 (2.20–5.36)***	7.95 (3.50–18.07)***	3.06 (1.15–8.13)*
Frequently vs. sometimes	2.43 (1.75–3.37)***	1.85 (1.25–2.73)***	2.45 (1.27–4.74)**	1.26 (0.59–2.70)

Note: *p < 0.05; **p < 0.01; ***p < 0.001. *OR* odds ratio.

[a] Multinomial logistic regression model, adjusted for victimization, parental education level, family structure and child psychopathology (the bullying question was excluded).

Table S10 Involvement in bullying at eight years of age as a bully or a bully-victim and committing any violent offenses by 31 years of age

	Males	Females
None, n (%)	1067 (42.3)	1843 (75.4)
Bully only, n (%)	419 (16.8)	204 (8.3)
Bully-victim, n (%)	1008 (40.4)	399 (16.3)
Bully only vs. no bully, HR (95% CI)[a]	2.14 (1.60−2.86)***	2.15 (1.12−4.15)*
Bully-victim vs. no bully, HR (95% CI)[a]	2.34 (1.85−2.96)***	2.09 (1.25−3.49)**
Bully-victim vs. bully only, HR (95% CI)[a]	1.09 (0.85−1.41)	0.97 (0.47−2.01)

Note: *$p < 0.05$; **$p < 0.01$; ***$p < 0.001$. *HR* hazard ratio.

[a] Cox regression model, unadjusted.
